# Editorial: Insights in Nutrition and Food Science Technology

**DOI:** 10.3389/fnut.2022.1129011

**Published:** 2023-01-09

**Authors:** Elena Ibañez, Alejandro Cifuentes

**Affiliations:** Foodomics Laboratory, Institute of Food Science Research (CIAL-CSIC), Madrid, Spain

**Keywords:** sustainability, food safety and quality, healthy diets, food production, Sustainable Development Goals (SDGs)

Nutrition and Food Science and Technology is facing important challenges nowadays, mainly related to the roadmap toward the achievement of the Sustainable Development Goals (SDGs) such as zero hunger (SDG-2), good health and wellbeing (SDG-3), responsible consumption and production (SDG-12), climate action (SDG-13), life below water (SDG-14), and life on land (SDG-15), among others. To be able to advance in these goals, sustainable food production systems are needed, while ensuring food safety (1) and more healthy diets (2). This Research Topic focuses on new insights, novel developments, current challenges, latest discoveries, recent advances, and future perspectives in the field of Nutrition and Food Science and Technology. The main goal of this special Research Topic is therefore, to shed light on the progress made in the past decade in the Nutrition and Food Science & Technology field, and on its future challenges to provide a thorough overview of the field. Based on all the above, the following 10 works were included in the current Research Topic. Specifically, two review papers were dedicated to describe the effects of microwave heating on the structure, properties, and functions of macromolecular nutrients in novel food (Deng et al.) and to revise the effect of resistant starch on non-alcoholic fatty liver disease *via* the gut-liver axis (Zhu et al.). One brief research report was devoted to analyse the accumulation of flavonoids in *Dendrobium moniliforme* (L.) Sw. (a valuable herbal crop) by the integration of metabolomic and transcriptomic approaches (Yuan et al.). The other seven papers were published as original research contributions and were focused on **(**i) the integrated analysis of metabolome and transcriptome data for uncovering flavonoid components of *Zanthoxylum bungeanum* Maxim leaves under drought stress (Hu et al.); **(**ii) the integrated metabolomic and transcriptomic analyses to reveal novel insights of anthocyanin biosynthesis on color formation in cassava tuberous roots (Fu et al.); **(**iii) the identification of iron and zinc responsive genes in pearl millet using genome-wide RNA-sequencing approach (Goud et al.); **(**iv) the identification and analysis of major flavor compounds in radish taproots by widely targeted metabolomics (Mei et al.); **(**v) the development of a new indicator of overeating saturated fat based on serum fatty acids and amino acids and its association with incidence of type 2 diabetes using two randomized controlled feeding trials and a prospective study (Wei et al.); **(**vi) the optimization of the ultrasound-assisted extraction of naturally occurring glucosinolates from by-products of *Camelina sativa* L. and their effect on human colorectal cancer cells (Pagliari et al.) and **(**vii) the investigation of the molecular mechanisms of flavonoid accumulation in germinating common bean (*Phaseolus vulgaris*) under salt stress (Zhang et al.). We hope this article collection may help, inform and provide direction and guidance to many colleagues working in this hot field of research.

## Author contributions

EI and AC acted as editors of all the manuscript submitted to the Research Topic *Insights in Nutrition and Food Science Technology*. AC supervised and wrote the editorial article. EI reviewed the final version of the editorial article. Both authors approved the final version of the manuscript for publication.
